# The Impact of Tooth Mobility and Furcation Involvement on Tooth Loss: A Retrospective Cohort Study

**DOI:** 10.3390/healthcare14081070

**Published:** 2026-04-17

**Authors:** Georgios S. Chatzopoulos, Larry F. Wolff

**Affiliations:** 1 Division of Periodontology, School of Dentistry, University of Minnesota, Minneapolis, MN 55455, USA; wolff001@umn.edu; 2 Faculty of Dentistry, Health Sciences, Aristotle University of Thessaloniki, 54124 Thessaloniki, Greece

**Keywords:** tooth loss, furcation involvement, tooth mobility, periodontitis, risk factors, prognosis, risk modeling, EHR-based study

## Abstract

**Background/Objectives**: The aim of this study was to analyze and quantify the independent effects of tooth mobility and furcation involvement on the probability of tooth loss in a large patient cohort, after controlling for patient-level confounding factors. **Methods**: This retrospective cohort study utilized data from 16,756 patients. Primary predictors were tooth mobility and furcation involvement. The primary outcome was tooth loss. A multivariable logistic regression model, adjusting for confounders (age, gender, smoking, diabetes), was developed to calculate Odds Ratios (OR) and 95% confidence intervals (CI). **Results**: A significant dose–response relationship was observed between the severity of both mobility and furcation involvement and the rate of tooth loss (*p* < 0.001). After multivariable adjustment, both remained statistically significant predictors (*p* < 0.001). Compared to no mobility, the odds of tooth loss for Class 3 mobility were 3.99 times higher (OR = 3.99; 95% CI: 3.58–4.45). Compared to no furcation involvement, the odds for Grade 3 involvement were 2.50 times higher (OR = 2.50; 95% CI: 2.19–2.85). Diabetes, smoking, male gender, and increasing age were also significant risk factors. **Conclusions**: Tooth mobility and furcation involvement are independent predictors of future tooth loss. These findings highlight the critical importance of thoroughly assessing mobility and furcation defects for patient risk assessment, prognosis, and treatment planning.

## 1. Introduction

Periodontitis is a chronic inflammatory disease characterized by the progressive destruction of the tooth-supporting tissues, including the alveolar bone and periodontal ligament [1]. It represents a profound global public health burden; recent epidemiological data (e.g., the Global Burden of Disease study) indicates that severe periodontitis affects approximately 11% to 14% of the global adult population, representing over 1 billion cases worldwide [2]. The pathogenesis of the disease is multifactorial, driven by a dysbiotic microbial biofilm but heavily modified by established systemic and environmental risk factors. Chief among these are smoking and poorly controlled diabetes mellitus, alongside genetic susceptibility, advancing age, poor oral hygiene, and socio-economic determinants [3]. If left untreated, this progressive attachment loss ultimately leads to tooth loss, compromising oral function, aesthetics, and patient quality of life [4].

As periodontitis progresses, it frequently culminates in specific anatomical and functional deficits, most notably furcation involvement and tooth mobility. Furcation defects develop when inflammatory alveolar bone resorption extends apically beyond the root trunk of multirooted teeth, exposing the complex interradicular space. The development of these defects is frequently accelerated by local anatomical predisposing factors—such as short root trunks, cervical enamel projections, enamel pearls, and deep root concavities—which harbor pathogenic biofilms that are highly inaccessible to both professional debridement and patient-led plaque control [5]. Conversely, clinical tooth mobility arises primarily from the quantitative loss of alveolar bone support. However, this mobility is often significantly amplified by qualitative changes within the periodontium, such as the widening of the periodontal ligament space due to secondary occlusal trauma, as well as the loss of tissue rigidity caused by active periodontal inflammation.

Previous meta-analyses have confirmed that molars with furcation involvement have a substantially higher risk of being lost compared to those without, even when patients are enrolled in supportive periodontal therapy [6,7]. This risk appears to increase with the severity of the furcation defect. Similarly, tooth mobility, which reflects the loss of alveolar bone support, is another critical clinical sign linked to a poorer prognosis.

While the relationships between periodontitis, mobility, and furcation involvement are established, the need remains to rigorously quantify their impact, especially after accounting for other patient-level influences. This study’s added value resides in providing quantified, independent risk data from a large, contemporary retrospective cohort using a unified multivariable logistic regression model. This approach isolates the contribution of mobility and furcation involvement while simultaneously adjusting for systemic confounders like age, gender, smoking, and diabetes, thereby refining the understanding of these risk factors. This study, therefore, aimed to analyze the independent and combined effects of baseline tooth mobility and furcation involvement on the probability of tooth loss in a large patient cohort.

## 2. Materials and Methods

This retrospective cohort study utilized de-identified data from the BigMouth Dental Data Repository. This multi-center network aggregates real-world clinical information from the electronic health records (EHRs) of nine participating U.S. university dental clinics. The patient population is diverse, consisting of individuals seeking comprehensive care at academic dental institutions, who often present with a wide range of dental conditions and systemic comorbidities. The repository provides longitudinal data, including patient demographics, self-reported medical/dental histories, full periodontal charting (e.g., probing depths, attachment loss, furcation measurements), and all treatments coded with the ADA’s Current Dental Terminology (CDT). This real-world data facilitates the analysis of clinical outcomes under routine practice conditions across a broad and varied patient population.

The study protocol (STUDY00016576) was reviewed by the University of Minnesota’s Institutional Review Board, which granted a waiver of approval, determining that the project did not constitute human subjects research under federal definitions. Ethical clearance was also obtained from the BigMouth Consortium for Oral Health Research and Informatics clinical review committee. The research was conducted in full accordance with the ethical principles outlined in the Helsinki Declaration of 1975, as revised in 2013.

The inclusion criteria for this study were: (1) adult patients (≥18 years of age); (2) patients with at least one recorded follow-up visit following their initial comprehensive or periodic periodontal evaluation; and (3) complete baseline periodontal charting that included assessments of both tooth mobility and furcation involvement. Patients were explicitly excluded from the final analysis if they met any of the following criteria: (1) age strictly under 18 years at baseline; (2) incomplete demographic data or medical history regarding smoking or diabetes; (3) incomplete baseline periodontal charting (i.e., missing scores for mobility or furcation involvement); or (4) a total follow-up period of less than 6 months between their baseline examination and their final recorded visit or tooth extraction. The follow-up period was defined as the time (in months) from the baseline periodontal charting to the date of the last recorded dental visit or the date of tooth extraction.

The patient population was diverse, encompassing individuals from various racial and ethnic backgrounds. The primary predictors of interest were baseline tooth mobility and baseline furcation involvement. Mobility was scored on a scale of 0 (no mobility) to 3 (severe mobility) [8]. Furcation involvement was also graded on a scale of 0 (no involvement) to 4 (through-and-through visible involvement) [9]. The primary outcome was tooth loss during the follow-up period. Confounding variables included in the analysis were patient age, gender, smoking status, and a diagnosis of diabetes.

To ensure a baseline level of standardization across the multicenter network, all participating institutions utilize standardized electronic health record platforms with standardized periodontal charting modules. Furthermore, all dental procedures and diagnoses are universally mapped using the ADA’s Current Dental Terminology (CDT) coding system, ensuring uniform data extraction across different clinical sites.

### Statistical Analysis

A multivariable logistic regression model was developed to assess the association between the predictor variables and the outcome of tooth loss. This model allowed for the calculation of Odds Ratios (OR) and their corresponding 95% confidence intervals (CI) to determine the independent contribution of each factor while adjusting for the effects of confounders. Additionally, Chi-Squared tests were performed to evaluate the statistical significance of the association between the categorical predictors (mobility and furcation) and tooth loss. A *p*-value of < 0.05 was considered statistically significant.

Baseline tooth mobility and furcation involvement were treated as categorical variables in the model. Variables were created for each class/grade, utilizing Class 0 (no mobility) and Grade 0 (no furcation involvement) as the reference categories to isolate the specific risk associated with each increasing level of severity. Prior to finalizing the model, standard diagnostics were performed; multicollinearity was assessed using the Variance Inflation Factor (VIF), with all variables showing VIF values well below the standard threshold of 5, indicating no significant collinearity among the predictors.

While time-to-event analyses (e.g., Cox regression) are often utilized for longitudinal data, a multivariable logistic regression approach was selected for this study. In retrospective, multi-center EHR data, the precise date of tooth extraction is frequently imprecise, particularly if teeth are lost or extracted outside the participating university networks and only noted as missing at a subsequent visit. Therefore, assessing the cumulative odds of tooth loss as a binary outcome over the follow-up period provides a robust and reliable measure of risk.

## 3. Results

The study cohort consisted of 16,756 individuals (Table 1). The mean age was 52.8 ± 14.3 years and the mean follow-up period was 34.3 months. The majority of patients were female (54.2%). Key systemic conditions included a 9.0% prevalence of diabetes and a 25.4% prevalence of high blood pressure. Current smokers comprised 7.2% of the cohort. A clear, direct relationship was observed between the severity of both mobility and furcation involvement and the rate of tooth loss (Table 2 and Table 3). For mobility, the percentage of teeth lost increased from 13.4% for teeth with no mobility (Class 0) to 39.8% for teeth with severe mobility (Class 3). A similar trend was evident for furcation involvement. The rate of tooth loss increased from 14.1% in teeth with no furcation involvement (Grade 0) to 30.1% for teeth with Grade 3 furcation involvement. A Chi-Squared analysis confirmed that these associations were highly statistically significant (*p* < 0.001 for both mobility and furcation).

The multivariable logistic regression model to predict tooth loss is shown in Table 4. After adjusting for age, gender, smoking, and diabetes, both baseline mobility and furcation involvement remained significant independent predictors of tooth loss (*p* < 0.001).

- Tooth Mobility: Compared to teeth with no mobility (Class 0), the odds of tooth loss were 1.54 times higher for teeth with a mobility class of 1 (95% CI: 1.49–1.59, *p* < 0.001). The odds increased to 1.95 for a class of 2 (95% CI: 1.82–2.09, *p* < 0.001) and to 3.99 for a class of 3 (95% CI: 3.58–4.45, *p* < 0.001).
- Furcation Involvement: Compared to teeth with no furcation involvement (Grade 0), the odds of tooth loss were 1.25 times higher for teeth with a furcation grade of 1 (95% CI: 1.20–1.30, *p* < 0.001). The odds increased to 1.63 for a grade of 2 (95% CI: 1.53–1.74, *p* < 0.001) and to 2.50 for a grade of 3 (95% CI: 2.19–2.85, *p* < 0.001).
- Confounding Factors: Diabetes (OR = 1.30), smoking (OR = 1.22), male gender (OR = 1.15), and increasing age (OR = 1.01 per year) were also confirmed as independent risk factors for tooth loss, with all demonstrating *p* < 0.001.

## 4. Discussion

This large-scale, retrospective cohort study provides robust statistical evidence that both tooth mobility and furcation involvement are major, independent risk factors for tooth loss, even after controlling for key patient-level characteristics like age, gender, smoking, and diabetes. The findings not only confirm established clinical principles but also quantify the precise risk, demonstrating a clear “dose–response” relationship: as the severity of either mobility or furcation involvement increases, the odds of tooth loss rise significantly and incrementally.

The magnitude of risk identified in our cohort aligns strongly with, yet expands upon, the existing literature. For instance, a systematic review by Nibali et al. demonstrated that molars with furcation involvement had a relative risk of loss approximately two times higher than molars without furcation involvement [6]. Our multivariable model corroborates this, showing an OR of 1.63 for Grade 2 and 2.50 for Grade 3 defects, indicating that as the anatomical niche deepens, the efficacy of routine maintenance diminishes exponentially. Furthermore, while previous literature [7] frequently identifies baseline mobility as a predictor of tooth loss, our study uniquely quantifies the steep ‘dose–response’ trajectory of this risk in a modern, multi-center cohort. The nearly four-fold increase in odds of loss for Class 3 mobility underscores that severe loss of alveolar bone support—the underlying biological driver of mobility—frequently surpasses the limits of successful supportive periodontal therapy, highlighting the critical threshold between salvageable and hopeless prognoses.

The analysis also validated the independent impact of the confounding variables, which is consistent with broad epidemiological data. The finding that diabetes (OR = 1.30), current smoking (OR = 1.22), male gender (OR = 1.15), and increasing age (OR = 1.01 per year) are all independent risk factors for tooth loss supports the model’s validity and aligns with established literature on systemic risks for periodontal disease progression [10,11,12].

From a clinical perspective, these findings are immediately applicable. They provide the quantitative evidence to support and refine tooth-specific prognostication, moving beyond subjective assessments.

- Patient Communication: Clinicians can use this data to more effectively communicate risk. Explaining that a tooth with Class 3 mobility is “nearly four times more likely to be lost” can be a tool for patient education and shared decision-making regarding treatment.
- Treatment Planning: The high, graded risk underscores the necessity of early intervention. The fact that even Class 1 mobility (OR = 1.54) and Grade 1 furcation (OR = 1.25) confer a significant increase in risk highlights the need to manage these issues before they become severe. For teeth with high-grade mobility and furcation involvement, this data can help guide clinicians and patients in deciding between complex, costly regenerative efforts versus more predictable options like extraction and replacement.

For teeth with high-grade mobility (Class 3) and severe furcation involvement (Grade 3), the exponential increase in risk establishes a clear clinical threshold. When these local factors are compounded by systemic risks (e.g., poorly controlled diabetes or smoking), the predictability of retention drops precipitously. In such scenarios, the data strongly supports favoring strategic extraction and site preservation over complex, costly, and ultimately unpredictable regenerative efforts, thereby sparing the patient from protracted, futile therapies.

- Risk Assessment: These findings emphasize that a thorough assessment of mobility and furcations is a non-negotiable part of a comprehensive periodontal exam. These factors must be considered key components when formulating a tooth’s prognosis.

It should be noted that the overall incidence of tooth loss in this cohort (ranging from 13.4% to nearly 40% for severely compromised teeth) appears elevated compared to private practice settings. This is largely attributable to the nature of the BigMouth repository, which aggregates data from university-based academic dental centers. These institutions frequently serve as referral centers for patients with advanced, complex, or historically neglected periodontal conditions, resulting in a higher baseline prevalence of ‘hopeless’ teeth slated for extraction shortly after initial examination.

### 4.1. Comparison with Previous Research

The magnitude of risk identified in our cohort must be contextualized within the broader literature on long-term periodontal prognosis and tooth survival. Extensive longitudinal studies consistently demonstrate that periodontal therapy combined with regular supportive periodontal care (SPC) is highly effective in preserving teeth, yielding extremely low annual tooth loss rates (0.04 to 0.15 teeth per patient per year) and long-term retention rates of 90% to 95% over 10 to 18 years [13,14,15,16]. In fact, a 30-year follow-up study reported an overall tooth loss rate of only 5.1% during supportive care [13], while a comprehensive 10-year meta-analysis confirmed an 89% survival rate for periodontally treated posterior teeth [17]. These favorable outcomes starkly contrast with the natural history of untreated periodontitis, which frequently culminates in severe tooth mortality and edentulism [18]. However, realizing these high survival rates requires strict adherence to maintenance protocols; erratic compliance increases the risk of tooth loss by 5.6 times [19], and insufficient maintenance visits significantly elevate the risk of tooth loss, particularly for patients with advanced disease stages [20].

Within this framework of long-term survival, our findings regarding baseline tooth mobility and furcation involvement strongly align with established prognostic models. Previous meta-analyses and retrospective studies consistently identify tooth-level factors—such as Grade III furcation involvement, increased mobility, and molar tooth type—as critical predictors of localized failure despite maintenance [7,14,15]. Furthermore, the independent risks posed by patient-level confounders such as diabetes, smoking, and advanced age in our model correspond directly with well-documented risks associated with systemic disease and advanced periodontitis staging [7,21]. While achieving complete disease resolution and strict stability is relatively rare [22], most patients undergoing SPC successfully retain the majority of their dentition over extended periods, with clinical improvements sustained over decades [15,23]. Therefore, our quantification of the steep, dose–response risk associated with severe mobility and furcation involvement highlights that these specific clinical signs represent critical prognostic thresholds. When these anatomical and structural deficits are present, routine maintenance may no longer be sufficient to prevent tooth loss, warranting early, targeted site-specific interventions or strategic extractions.

### 4.2. Strengths and Limitations

The primary strength of this study is its large, diverse, real-world cohort of 16,756 patients sourced from a multi-center repository. The “BigMouth” dataset enhances the generalizability of the findings to typical practice conditions, which is an advantage over traditional, highly controlled clinical trials. The use of a multivariable logistic regression model was a key methodological strength, allowing for the successful isolation of the independent prognostic value of mobility and furcation.

Despite these strengths, the study has several limitations inherent to its retrospective design. The data was aggregated from nine different university clinics, which introduces the potential for significant inter-examiner variability in the clinical scoring of mobility and furcation grades, as provider calibration could not be verified. Furthermore, while the multivariable model controlled for key systemic factors like diabetes and smoking, it could not account for other critical, unmeasured confounders. These include patient-specific factors (e.g., oral hygiene practices, adherence to supportive periodontal therapy), disease-specific variables (e.g., the specific staging and grading of periodontitis, glycemic control in diabetics), and treatment-specific interventions (e.g., whether a tooth received surgical vs. non-surgical therapy, or if mobile teeth were splinted). Additional limitations include the exclusion of Grade 4 furcation defects from the regression model (Table 4). Although only 17 such teeth were present in the dataset (Table 3), their exclusion means the calculated risk for Grade 3 involvement (OR = 2.50) may be an underestimation of the true risk posed by end-stage furcation involvement. Moreover, the use of logistic regression, while justified by the nature of the EHR data, evaluates cumulative risk rather than precise time-to-event survival dynamics.

In addition, while the multivariable model controlled for key systemic factors available within the EHR (diabetes and smoking), it was limited by the unavailability of other critical confounding variables. Retrospective database analyses are inherently constrained by data completeness. Consequently, we were unable to adjust for socioeconomic status, individual oral hygiene indices (e.g., plaque and bleeding scores), the specific staging and grading of the patients’ periodontitis, or the exact nature of the periodontal therapy received (surgical vs. non-surgical). The inability to control for these unmeasured variables means the reported Odds Ratios may partially reflect these underlying, uncaptured disease severity and compliance factors.

Future research using a prospective design would be valuable to confirm these findings while controlling for treatment interventions and patient adherence. Nonetheless, this study provides quantitative data affirming that tooth mobility and furcation involvement are cornerstone prognostic factors in periodontal disease management.

## 5. Conclusions

This large-scale retrospective analysis confirms that tooth mobility and furcation involvement are independent predictors of future tooth loss. The risk increases substantially in a dose–response manner with the severity of these clinical signs. Beyond merely confirming established tenets, this study quantifies these risks within a diverse, real-world cohort, demonstrating that severe mobility and advanced furcation defects represent critical prognostic thresholds. When these anatomical and structural deficits are present, routine maintenance may no longer be sufficient to prevent tooth loss. Therefore, the thorough assessment of these factors is essential for accurate risk stratification, enabling clinicians and patients to make evidence-based decisions regarding strategic tooth retention, targeted regenerative therapies, or extraction and prosthetic replacement.

## Figures and Tables

**Table 1:** Characteristics of the included cohort (*n* = 16,756).

Characteristic	Category	Value
Age (years)	Mean (SD)	52.8 (14.3)
	Range (Min–Max)	18–93
Gender	Female	9083 (54.2%)
	Male	7628 (45.5%)
	UNANSWERED	45 (0.3%)
Race	White	5829 (34.8%)
	Hispanic or Latino (Race)	4040 (24.1%)
	Black or African American	2800 (16.7%)
	Not Specified	1530 (9.1%)
	Asian	1351 (8.1%)
	Some Other Race	1138 (6.8%)
	Am. Indian or Alaskan Native	57 (0.3%)
	Pacific Islander	11 (0.1%)
Ethnicity	Non-Hispanic	7622 (45.5%)
	Not Specified	5055 (30.2%)
	Hispanic	3193 (19.1%)
	Others	886 (5.3%)
Systemic Factors & Smoking	Cigarettes	1202 (7.2%)
	Diabetes	1509 (9.0%)
	High Blood Pressure	4254 (25.4%)
	Cardiovascular Heart Problem	1260 (7.5%)

**Table 2:** Tooth loss by tooth mobility class.

Mobility Class	Total Teeth	Teeth Lost	Percent Lost
0	162,267	21,767	13.4%
1	29,611	5851	19.8%
2	4646	1125	24.2%
3	1093	435	39.8%

Mobility Class definitions: Class 0 = physiologic mobility; Class 1 = slightly more than physiologic mobility (<1 mm horizontally); Class 2 = moderately more than physiologic mobility (1–2 mm horizontally); Class 3 = severe mobility (>2 mm horizontally and/or vertical depression).

**Table 3:** Tooth loss by furcation involvement grade.

Furcation Grade	Total Teeth	Teeth Lost	Percent Lost
0	171,223	24,202	14.1%
1	17,729	3016	17.0%
2	7706	1670	21.7%
3	942	284	30.1%
4	17	6	35.3%

Furcation Grade definition: Grade 0 = no involvement; Grade 1 = horizontal loss of periodontal tissue support <3 mm; Grade 2 = horizontal loss of support >3 mm but not encompassing the total width of the furcation; Grade 3 = through-and-through destruction; Grade 4 = through-and-through destruction with clinical visibility due to gingival recession.

**Table 4:** Multivariable logistic regression model to predict tooth loss.

Predictor	Category	Odds Ratio (OR)	95% Confidence Interval	*p*-Value
Baseline Mobility	Class 1 (vs. 0)	1.54	1.49–1.59	<0.001
	Class 2 (vs. 0)	1.95	1.82–2.09	<0.001
	Class 3 (vs. 0)	3.99	3.58–4.45	<0.001
Baseline Furcation	Grade 1 (vs. 0)	1.25	1.20–1.30	<0.001
	Grade 2 (vs. 0)	1.63	1.53–1.74	<0.001
	Grade 3 (vs. 0)	2.50	2.19–2.85	<0.001
Diabetes	Yes (vs. No)	1.30	1.22–1.38	<0.001
Smoking	Yes (vs. No)	1.22	1.13–1.32	<0.001
Gender	Male (vs. Female)	1.15	1.12–1.19	<0.001
Patient Age	(per year)	1.01	1.01–1.01	<0.001

Mobility Class definitions: Class 0 = physiologic mobility; Class 1 = slightly more than physiologic mobility (<1 mm horizontally); Class 2 = moderately more than physiologic mobility (1–2 mm horizontally); Class 3 = severe mobility (>2 mm horizontally and/or vertical depression). Furcation Grade definition: Grade 0 = no involvement; Grade 1 = horizontal loss of periodontal tissue support <3 mm; Grade 2 = horizontal loss of support >3 mm but not encompassing the total width of the furcation; Grade 3 = through-and-through destruction; Grade 4 = through-and-through destruction with clinical visibility due to gingival recession.

## Data Availability

The data presented in this study are available on request from the corresponding author due to privacy/ethical restrictions. The data are not publicly available as they contain information that could compromise the privacy of research participants. The dataset contains sensitive personal information, and participant consent did not include public data sharing.
